# Predictive significance of inflammatory markers in the survival of older Indian patients with cancer: a single-center prospective analysis

**DOI:** 10.3332/ecancer.2024.1746

**Published:** 2024-08-22

**Authors:** Abhijith Rajaram Rao, Vanita Noronha, Anant Ramaswamy, Anbarasan Sekar, Anita Kumar, Anupa Pillai, Shreya Gattani, Arshiya Sehgal, Sharath Kumar, Renita Castelino, Ratan Dhekale, Jyoti Krishnamurthy, Sarika Mahajan, Anuradha Daptardar, Lekhika Sonkusare, Jayita Deodhar, Nabila Ansari, Manjusha Vagal, Purabi Mahajan, Shivshankar Timmanpyati, Manjunath Nookala, Ankita Chitre, Akhil Kapoor, Vikram Gota, Shripad Banavali, Rajendra A Badwe, Kumar Prabhash

**Affiliations:** 1 Department of Geriatric Medicine, All India Institute of Medical Science, New Delhi 110029, India; 2 Department of Medical Oncology, Tata Memorial Centre, Mumbai 400012, India; 3 Department of Clinical Pharmacology, Advanced Centre for Treatment Research and Education in Cancer, Kharghar, Navai Mumbai 410210, India; 4 Department of Physiotherapy, Tata Memorial Centre, Homi Bhabha National Institute, Mumbai 400012, India; 5 Department of Psycho-oncology, Tata Memorial Centre, Homi Bhabha National Institute, Mumbai 400012, India; 6 Department of Occupational Therapy, Tata Memorial Centre, Homi Bhabha National Institute, Mumbai 400012, India; 7 Department of Digestive Diseases and Clinical Nutrition, Tata Memorial Centre, Homi Bhabha National Institute, Mumbai 400012, India; 8 Department of Physiotherapy, Mahamana Pandit Madan Mohan Malviya Cancer Center, Homi Bhabha Cancer Hospital, Varanasi 221005, India; 9 Department of Medical Oncology, Mahamana Pandit Madan Mohan Malviya Cancer Center, Homi Bhabha Cancer Hospital, Varanasi 221005, India; 10 Department of Surgical Oncology, Tata Memorial Centre, Homi Bhabha National Institute, Mumbai 400012, India

**Keywords:** inflammatory markers, NLR, LMR, PLR, Indian older adults, prognostic marker

## Abstract

**Aim:**

To evaluate the prognostic impact of neutrophil-lymphocyte ratio (NLR), platelet-lymphocyte ratio (PLR) and lymphocyte-monocyte ratio (LMR) on overall survival (OS) among Indian older patients with cancer.

**Methods:**

This observational study was conducted in the geriatric oncology clinic of Tata Memorial Hospital (India). We included all patients who underwent a geriatric assessment (GA) and had a complete blood count available for analysis. The NLR was dichotomized at 3.5, PLR and LMR at the median. Our primary study outcome was OS.

**Results:**

Between June 2018 and November 2021, 786 patients were enrolled (median age: 69 years). The most common primary tumour was lung (308, 39.5%), followed by gastrointestinal (261, 33.5%). Metastatic disease was present in 54.3% of patients. Univariate analysis revealed that patients with NLR >3.5 had shorter OS (9.1 months) than NLR <3.5 (15.7 months) (HR: 1.56). Similarly, patients with PLR >183.5 had reduced OS (9.3 months) compared to PLR <183.5 (16.6 months) (HR: 1.56). Conversely, patients with LMR >3.1 showed better OS (14.2) compared to LMR <3.1 (9.8 months) (HR: 0.74). After adjusting for age, performance status, primary tumour, metastatic status and GA-derived factors (function, nutrition and cognition), NLR (HR: 1.25, 95%CI: 1.03–1.52), PLR (HR: 1.34, 95%CI: 1.11–1.63) and LMR (HR: 0.79, 95%CI: 0.65–0.95) were associated with OS.

**Conclusion:**

In our study of older cancer patients, we identified three key inflammatory markers (NLR >3.5, PLR >183.5, LMR <3.1) as strong predictors of poor OS. These markers remain predictive even after accounting for traditional prognostic factors and GA-derived scales.

## Background

Ageing and inflammation are closely related, leading to the expression ‘inflammaging’ [1]. Numerous age-related conditions, including cognitive impairment, depression and sarcopenia, share an inflammatory pathophysiological mechanism [2]. Inflammatory markers have been identified as predictors of unfavourable health outcomes, such as functional decline and death [3]. The value of prognostic models to improve the categorisation of patient risk by incorporating information from multiple pretreatment factors is widely accepted in oncology [4, 5].

Cancer is also intimately associated with inflammation [6, 7]. Systemic inflammatory markers of including C-reactive protein (CRP), albumin, neutrophil, lymphocyte, neutrophil-lymphocyte ratio (NLR), platelet-lymphocyte ratio (PLR), lymphocyte-monocyte ratio (LMR), Glasgow prognostic score (GPS), modified GPS (mGPS) and interleukin-6 (IL-6) are studied as an indicator of inflammatory response, to identify new prognostic factors for cancer [8–10].

Among these, NLR, PLR and LMR are inexpensive to test and routinely measured in day-to-day oncological practice, potentially providing readily available objective information to help oncologists estimate patient prognosis. These have been shown to have robust prognostic value, independent of traditional factors, such as age, performance stage and cancer stage [11–13]. There is no data on the utility of these prognostic markers from the Indian subcontinent. In this study, we evaluate the prognostic impact of NLR, LMR and PLR on overall survival (OS) among Indian older patients with cancer.

## Materials and methods

### General study details

This observational study was conducted within the geriatric oncology clinic at Tata Memorial Hospital, India, from June 2018 to November 2021. The clinic, established in June 2018, is staffed by a multidisciplinary team including medical oncologists, geriatricians, physiotherapists, occupational therapists, clinical pharmacologists, psychologists, dieticians and social workers [14]. All patients underwent a geriatric assessment (GA). Institutional Ethics Committee approval was obtained in March 2020 (Project Number 900596), with a waiver of written informed consent for patients assessed before this date. Subsequently, enrolled patients provided written informed consent. The declaration of Helsinki’s tenets and Good Clinical Practice Guidelines were followed in the conduct of the study. It was registered with the Clinical Trial Registry-India (CTRI/2020/04/024675). No external funding was utilised.

### Participants

GA is performed on cancer patients 60 years of age and older who have been diagnosed and are scheduled for systemic therapy. We included all patients who underwent a GA and had a complete blood count available for analysis.

### Variables

Our primary objective was to identify if inflammatory markers, such as NLR, PLR and LMR, were associated with OS among older patients with cancer.

### Study methodology

Pretreatment neutrophil, lymphocyte, platelet and monocyte counts were collected from electronic medical records. The independent factors that were evaluated were the LMR, PLR and NLR. The NLR was dichotomized at 3.5, the upper boundary of a 95% confidence interval observed in a healthy adult population [15]. Since PLR and LMR’s reference values were not yet determined, they were dichotomized at the median.

As part of GA, patients underwent a comprehensive evaluation of geriatric non-oncological domains including function, nutrition, cognition, falls, comorbidities, medications, psychological status and social support. Our analysis revealed that function, nutrition and cognition significantly impacted survival, prompting their inclusion in this study.

The function was assessed using Katz activities of daily living (ADL) [16], Lawton instrumental activities of daily living (IADL) [17] and Timed-up-and-Go [18]. The impaired function was defined as an ADL score less than 6, an IADL score less than 5 for men or 8 for women, or a Timed-up-and-Go (TUG) time greater than 10 seconds.

Nutritional status was evaluated through body mass index (BMI), unintentional weight loss within the past 3 months and the Mini-Nutritional Assessment [19]. Individuals were classified as having poor nutrition if their BMI was less than 18.5 kg/m^2^, their unintended weight loss was more than 10%, or their Mini-Nutritional Assessment (MNA) score was less than 24 [20].

The Mini-Mental Status Examination [21] was utilised to evaluate cognitive abilities in literate patients, while the Hindi Mental Status Examination [22] was utilised for illiterate patients. A score below 24 on either scale indicated cognitive impairment.

The Eastern Cooperative Oncology Group Performance Status (ECOG PS) was derived from the GA results.

### Survival

Survival status was determined through telephone contact with all patients between 3 November and 20 December 2022. Participants who could not be reached or had not visited the hospital within the previous 3 months were considered lost to follow-up. The last known date of survival, as determined by the most recent hospital visit or telephone contact, was used as the censoring date. From the date of the GA to the date of death from any cause, the OS was computed.

### Statistical analysis

An a priori sample size calculation was not done. STATA version 14 (StataCorp. 2015. Stata Statistical Software: Release 14. College Station, TX: StataCorp LP) was used for all analyses. Descriptive statistics were employed to characterize the population’s baseline characteristics. The primary outcome was OS, which was measured from the date of undergoing the GA to death from any cause. Survival was estimated using the Kaplan-Meier method, and survival curves were compared using a log-rank test [23]. The median follow-up period was determined by the reverse Kaplan-Meir technique. The study employed multivariate Cox proportional hazards models to evaluate the independent impact of inflammatory markers on OS. Two multivariable models were built, a ‘traditional mode’ adjusting for age, ECOG PS, primary tumour and metastatic status and a ‘fully adjusted model’ adjusting for age, ECOG PS, primary tumour, metastatic status and GA-derived prognostic factors including, the domains of function, nutrition and cognition [24, 25].

## Results

### General patient details

Between June 2018 and November 2021, we evaluated 807 patients in the geriatric oncology clinic. Among them, 786 patients had complete blood count available. The median age was 69 (IQR: 65–73). 602 (76.6%) were male and 184 (23.4%) were female (Table 1). The most common primary tumour was lung (308, 39.5%), followed by gastrointestinal (261, 33.5%), head and neck (94, 12.1%) and genitourinary (86, 11.0%). Metastatic disease was present in 418 (54.3%) patients.

### Inflammatory markers

The median NLR, PLR and LMR were 3.4 (IQR: 2.4–5.2), 183.5 (IQR: 127.3–265.6) and 3.1 (IQR: 2.1–4.5), respectively (Table 1).

### Association of inflammatory markers with survival

**Figure 1.**
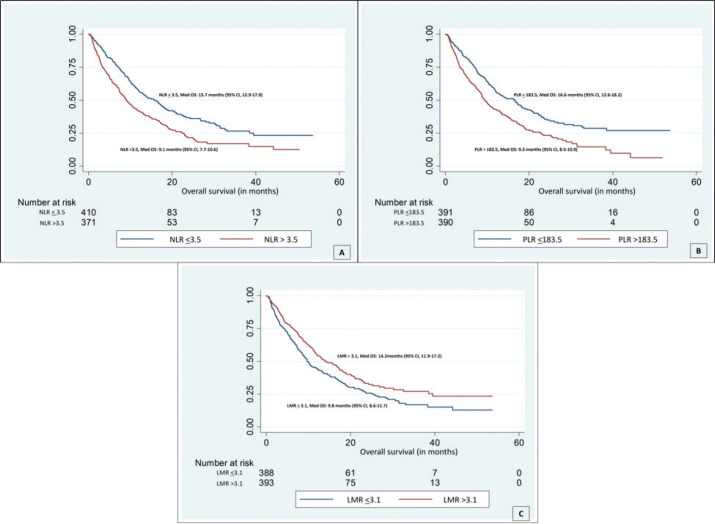
Kaplan-Meier curves displaying the estimated survival probability of patients with (a): NLR <3.5 (blue); median survival was 15.7 months and NLR >3.5 (red) with median survival of 9.1, *p *< 0.001; (b): PLR <183.5 (blue); median survival was 16.6 months and PLR >183.5 (red) with median survival of 9.3, *p *< 0.001; (c): PLR <183.5 (blue); median survival was 16.6 months and PLR >183.5 (red) with median survival of 9.3, *p *< 0.001.

The median follow-up period by reverse Kaplan-Meir technique was 21.5 months (95% CI 20.1–22.4). During the follow-up, 497 (63.2%) deaths had occurred and 60 (7.6%) were lost to follow-up. On univariate analysis, patients with NLR >3.5 had a poor OS (median OS: 9.1 months) compared to those with NLR <3.5 (median OS: 15.7 months) (HR: 1.56, 95% CI: 1.30–1.86, *p*-value: <0.001) (Figure 1). Patients with a PLR >183.5 had poor OS (median OS: 9.3 months) compared to those with PLR <183.5 (median OS: 16.6 months) (HR: 1.56, 95% CI: 1.31–1.87, *p*-value: <0.001) (Figure 1). Patients with an LMR >3.1 had a better OS (median OS: 14.2) compared to those with an LMR <3.1 (median OS: 9.8 months) (HR: 0.74, 95% CI: 0.62–0.89, *p*-value: 0.001) (Figure 1) (Table 2).

The Cox proportional hazards model was used for multivariate analysis (Table 3). The first model was adjusted for traditional prognostic factors such as age, ECOG PS, primary tumour and metastatic status. NLR (HR: 1.32, 95% CI: 1.09–1.59, *p*-value: 0.004), PLR (HR: 1.40, 95% CI: 1.17–1.68, *p*-value: <0.001) and LMR (HR: 0.78, 95% CI: 0.65–0.94, *p*-value: 0.009) were significantly associated with mortality. After adjusting for additional prognostic factors derived from GA (function, nutrition and cognition), NLR (HR: 1.25, 95% CI: 1.03–1.52, *p*-value: 0.023), PLR (HR: 1.34, 95% CI: 1.11–1.63, *p*-value: 0.003) and LMR (HR: 0.79, 95% CI: 0.65–0.95, *p*-value: 0.013) were associated with mortality.

## Discussion

In this outpatient-based study conducted among older patients with cancer, we found that inflammatory markers, such as NLR, PLR and LMR, were associated with OS in univariate and multivariate analyses. Notably, these inflammatory markers retained their prognostic significance independently of factors such as age, primary tumour location, metastatic status, performance status, functional assessments derived from GA, nutritional status and cognitive function results.

We found that an NLR >3.5 was significantly associated with poor OS (9.1 months) compared to patients with NLR <3.5 (15.7 months, *p* < 0.001). Even after accounting for additional variables such as functional and nutritional health as well as more conventional prognostic markers such as age, ECOG PS, initial tumour site and metastatic status, this association persisted. A meta-analysis encompassing 40,559 patients with solid tumours also reported a strong correlation between higher NLR and worse OS (HR: 1.81, *p* < 0.001) [26]. Furthermore, another systematic review and meta-analysis, comprising 41 cohort studies, demonstrated a significant link between elevated NLR (HR 1.60) and diminished survival in patients with gastric cancer [27]. It is worth noting that this meta-analysis did not exclusively focus on older cancer patients, with the mean age across the studies ranging from 52.5 to 69 years. Additionally, it is essential to acknowledge the ongoing debate concerning the appropriate cutoff value for NLR assessment. Some studies employ cutoffs based on medians, values determined through receiver-operating curves, or higher quartiles [15]. In our study, we utilised a predefined NLR cutoff based on the upper boundary of the 95% confidence interval in the healthy adult population [15].

The precise mechanism underlying the relationship between NLR and reduced survival in older cancer patients remains elusive. It is challenging to discern whether the elevated NLR results from cancer itself or the natural ageing processes. A prospective observational study conducted among older patients with cancer aged >70 years with solid malignant tumours, found that GPS was significantly associated with frailty (OR: 18.5) [28]. A reduction in physiologic reserve in several organ systems and heightened susceptibility are signs of frailty [29]. A low lymphocyte count is believed to be a marker of immunosenescence [30]. An elevated NLR could also reflect cancer-related inflammation, generating tumour-promoting microenvironment promoting cancer cell survival and proliferation [31]. There may be further explanations for the correlation between high NLR and poor OS, such as the secretion of hepatocyte growth factor and vascular endothelial growth factor by neutrophils [32, 33], and the function of lymphocytes in the humoral and cellular antitumour immune response [34, 35].

PLR has been identified as a valuable prognostic factor in several tumour types, including lung, colorectal and oesophagal cancers [36–38]. However, it is important to note that the association between PLR and prognosis is not consistently observed [39]. Patients with solid tumour cancer who were above 65 years old participated in this trial. Though PLR was significantly associated with OS in univariate analysis, this significance was lost when they adjusted for factors such as age, physician-rated Karnofsky performance status, cancer type, metastatic status and treatment intensity [39]. A systematic review and meta-analysis comparing 33 cohort studies with 8,215 patients, reported that elevated PLR was associated with reduced OS (HR: 1.45, 95% CI, 1.31–1.61, *p* < 0.001) [40]. The mechanism of the predictive value of blood PLR in cancer remains unclear. Elevated PLR indicates activation of transcription factors in inflammation response (nuclear factor-kB, hypoxia-inducible factor 1a, signal transducer and activator of transcription 3). These factors coordinate to produce tumour growth-promoting cytokines, including tumour necrosis factor-α, interleukin-1β and IL-6 [41, 42].

A low LMR was associated with poor OS among older patients with cancer. LMR is predictive of survival among patients undergoing surgery for colorectal [43], lung [44] and gastric cancer [45]. Systematic review and meta-analysis have reported that decreased pretreatment LMR in peripheral blood is associated with shorter OS in lung cancer (HR: 1.61, 95%CI: 1.45–1.79, *p*-value <0.01) [46] and pancreatic cancer (HR: 0.60, 95% CI: 0.50–0.71, *p*-value <0.001) [47]. Like NLR and PLR, the underlying molecular mechanisms of LMR are potentially complex and have not been fully elucidated. Lymphoid cells (T cells, B cells and mature dendritic cells) seem to generate and maintain local and systemic adaptive antitumour responses [47, 48]. Monocytes are important regulators favoring tumour invasion and metastasis, and their number negatively correlates with clinical outcomes [49].

## Strengths and limitations

Our study featured a substantial and diverse group of older patients with cancer, allowing for a more comprehensive analysis of the relationships between inflammatory markers and survival outcomes in this population. We conducted a rigorous multivariate analysis that considered conventional factors such as age, metastatic status and performance status and incorporated prognostic domains derived from GA. This approach revealed the independent predictive value of inflammatory markers for poor survival. By highlighting the independent prognostic significance of inflammatory markers, our study contributes valuable insights into the predictive factors for survival in older cancer patients. This information can guide clinicians in risk assessment and treatment planning.

Our study cohort encompassed a wide range of older patients with varying cancer types and stages. Because of this heterogeneity, there may be variation in the outcomes, and extrapolating our findings to certain subgroups of older cancer patients may be difficult. The study was conducted in a single healthcare center, potentially limiting the generalisability of our results to broader patient populations or other geographic regions. While our study focused on certain inflammatory markers, such as those mentioned, we did not include other potential prognostic markers such as GPS, mGPS, CRP or IL-6. The omission of these markers may limit the comprehensiveness of our findings, as they could have provided additional insights into survival prediction.

## Conclusion

In our study of older cancer patients, we identified three key inflammatory markers (NLR >3.5, PLR >183.5 and LMR <3.1) as strong predictors of poor OS. These markers remain predictive even after accounting for traditional prognostic factors and GA-derived scales. Combining these markers with GA variables can enhance prognostic accuracy. These easily accessible markers offer a practical tool for clinicians, aiding in more tailored care for older cancer patients.

## Conflicts of interest

The authors declare that they have no conflict of interest.

## Funding

No funding received.

## Patient consent

For patients enrolled before March 2020, consent was waivered by IEC. All Participants enrolled after March 2020 had given written informed consent.

## Ethical approval

Institutes Ethics Committee (IEC) approval was taken.

## Data availability

Data will be available on reasonable request from the corresponding author from the date of publication.

## Author contributions

All authors met the criteria for authorship as follows: study concept and design (Vanita Noronha, Anant Ramaswamy, Manjunath Nookala, Vikram Gota, Anuradha Daptardar, Jayita Deodhar, Manjusha Vagal, Shivshankar Timmanpyati, Akhil Kapoor, Shripad Banavali, Rajendra A Badwe and Kumar Prabhash), acquisition of data (Abhijith Rajaram Rao, Anita Kumar, Anupa Pillai, Shreya Gattani, Arshiya Sehgal, Sharath Kumar, Renita Castelino, Ratan Dhekle, Jyoti Krishnamurthy, Sarika Mahajan, Lekhika Sonkusare, Nabila Ansari, Purabi Mahajan and Ankita Chitre), analysis and interpretation of data (Abhijith Rajaram Rao, Vanita Noronha, Anant Ramaswamy and Kumar Prabhash) and preparation of manuscript (Abhijith Rajaram Rao, Vanita Noronha, Anant Ramaswamy and Kumar Prabhash). All authors approved the final manuscript. No unnamed contributor played a role in manuscript preparation.

## Figures and Tables

**Table 1.** Baseline characteristics of patients (*n* = 786).

Variables	No. of patients	Percentage (%)
Age (Median (IQR))	69	65–73
Sex		
Male	602	76.6
Female	184	23.4
BMI		
<18.5	153	19.5
18.5–22.9	334	42.6
23–24.9	133	17.0
>25	164	20.9
Primary tumour		
Lung	308	39.5
Gastrointestinal	261	33.5
Genito urinary	86	11.0
Head and neck	94	12.1
Others	30	3.9
Stage		
I	7	0.9
II	72	9.3
III	274	35.5
IV	418	54.3
ECOG PS		
0	53	6.8
1	420	53.9
2	228	29.2
3	79	10.1
Inflammatory markers	Median	IQR
Neutrophils (10^3^/mm^3^)	5.51	4.13–7.53
Lymphocyte (10^3^/mm^3^)	1.59	1.19–2.02
Monocyte (10^3^/mm^3^)	0.52	0.39–0.71
Platelet (10^3^/mm^3^)	289	215–374
NLR	3.4	2.4–5.2
<3.5	412	52.4
>3.5	374	47.6
PLR	183.5	127.3–265.6
<183.5	393	50.0
>183.5	393	50.0
LMR	3.1	2.1–4.5
<3.1	392	49.9
>3.1	394	40.1
Geriatric assessment results		
Function (*n* = 771)		
Normal	314	40.7
Impaired	457	59.3
Nutrition (*n* = 784)		
Normal	258	32.9
Impaired	526	67.1
Cognition (*n* = 766)		
Normal	653	85.3
Impaired	113	14.7

BMI: body mass index, PS: performance status, NLR: neutrophil-lymphocyte ratio, PLR: platelet-lymphocyte ratio, LMR: lymphocyte-monocyte ratio, ECOG PS: European Cooperative Oncology Group Performance Status

**Table 2.** Univariate survival analysis.

Variables		Survival (months)	HR	95% CI	*p*-value
Cellular markers of inflammation					
NLR	<3.5	15.7 (12.9–17.9)	1 (reference)		<0.001
	>3.5	9.1 (7.7–10.6)	1.56	1.30–1.86	
PLR	<183.5	16.6 (12.6–18.2)	1 (reference)		<0.001
	>183.5	9.3 (8.5–10.9)	1.56	1.31–1.87	
LMR	<3.1	9.8 (8.6–11.7)	1 (reference)		0.001
	>3.1	14.2 (11.9–17.2)	0.74	0.62–0.89	
Traditional prognostic factors					
Age			1.02	1.01–1.04	0.001
ECOG PS					
0			1 (reference)		
1			1.36	0.92–2.02	0.125
2			2.12	1.41–3.18	<0.001
3			2.96	1.89–4.64	<0.001
Primary tumour					
Other			1 (reference)		
Lung			1.89	1.12–3.19	0.017
Gastrointestinal			1.38	0.81–2.35	0.23
Genito urinary			0.91	0.50–1.65	0.763
Head and neck			1.61	0.91–2.93	0.101
Stage					
I–III			1 (reference)		
IV			1.50	1.25–1.80	<0.001
Additional prognostic factors					
Function domain					
Normal			1 (reference)		
Impaired			1.52	1.26–1.83	<0.001
Nutrition domain					
Normal			1 (reference)		
Impaired			1.54	1.27–1.88	<0.001
Cognition domain					
Normal			1 (reference)		
Impaired			1.59	1.26–2.01	<0.001

PS: performance status, NLR: neutrophil-lymphocyte ratio, PLR: platelet-lymphocyte ratio, LMR: lymphocyte-monocyte ratio

**Table 3.** Multivariable survival analysis.

Variables	Adjusted for traditional factors^a^		Adjusted for additional factors^b^	
	HR (95% CI)	*p*-value	HR (95% CI)	*p*-value
NLR	1.32 (1.09–1.59)	0.004	1.25 (1.03–1.52)	0.023
PLR	1.40 (1.17–1.68)	<0.001	1.34 (1.10–1.63)	0.003
LMR	0.78 (0.65–0.94)	0.009	0.79 (0.65–0.95)	0.013

a Adjusted for age, ECOG PS, Primary tumour, metastasis

b Adjusted for age, ECOG PS, Primary tumour, metastasis, function, nutrition and cognition domains on geriatric assessment

NLR: neutrophil-lymphocyte ratio, PLR: platelet-lymphocyte ratio, LMR: lymphocyte-monocyte ratio
